# Role of Cyanobacteria in the assembly and dynamics of microbial communities on glacier surfaces

**DOI:** 10.1016/j.isci.2025.112061

**Published:** 2025-02-18

**Authors:** Yeteng Xu, Yang Liu, Tuo Chen, Shijin Wang, Guangxiu Liu, Gaosen Zhang, Wei Zhang, Minghui Wu, Ximing Chen, Binglin Zhang

**Affiliations:** 1 Yulong Snow Station of Cryosphere and Sustainable Development, State Key Laboratory of Cryospheric Science and Frozen Soil Engineering, Northwest Institute of Eco- Environment and Resources, Chinese Academy of Sciences, Lanzhou 730000, China; 2 University of Chinese Academy of Sciences, Beijing 100049, China; 3 Key Laboratory of Extreme Environmental Microbial Resources and Engineering of Gansu Province, Lanzhou 730000, China; 4 Key Laboratory of Desert and Desertification, Northwest Institute of Eco-Environment and Resources, Chinese Academy of Sciences, Lanzhou 730000, China; 5 Key Laboratory of Soil Ecology and Health in Universities of Yunnan Province, School of Ecology and Environmental Science, Yunnan University, Kunming 650091, China

**Keywords:** Earth sciences, Biogeoscience, Microbiology

## Abstract

Glacier surface habitats are dynamic ecosystems that respond to local climatic and thermal changes, although the assembly mechanisms of microbial communities in these environments remain unclear. This study examined microbial communities on the surface of Baishui Glacier No. 1 across the accumulation, the intense melt, and the late melt periods. The absolute abundance of Cyanobacteria increased significantly, becoming the most abundant phylum by the end of the melt period. Cyanobacteria were strongly associated with other local microorganisms, especially in community structure, community assembly, and co-occurrence networks. The correlations between Cyanobacteria and other microorganisms shifted from predominantly mutualistic interactions, to being predominantly competitive interactions, and finally to mutualistic interactions with a portion of the community. Additionally, Cyanobacteria abundance positively correlated with nitrogen metabolism multifunctionality in other microorganisms, indicating a potential link between Cyanobacteria and nitrogen cycling. These findings provide new insights into microbial community dynamics and survival strategies on glacier surfaces.

## Highlights

• Cyanobacteria dominate microbial communities in glacier surface snow-ice habitats
• Cyanobacteria strongly correlate with local microbial community diversity and assembly
• Cyanobacteria significantly contribute to the stability of local microbial networks
• Cyanobacteria correlate with nitrogen metabolism multifunctionality of local microbes

## Graphical abstract

**Figure:**
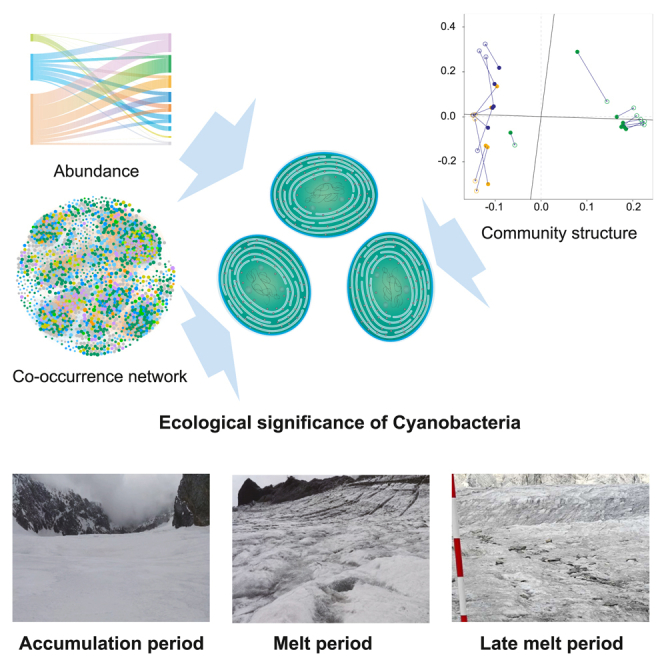

## Introduction

Glacial habitats present unique environmental conditions where microbial communities persist on glacier surfaces and significantly impact biogeochemistry.^1^^,^^2^ Microbial life in these habitats faces extreme challenges such as low temperatures, limited nutrient availability, high UV radiation, and scarcity of liquid water. These harsh conditions exert substantial evolutionary pressures, resulting in unique biodiversity in glacier habitats.^3^^,^^4^ The study of these specialized microbial consortia not only furthers our understanding of biogeography^5^ and ecosystem function in cryosphere^1^ but also reveals information about potential extraterrestrial life, biogeochemical cycles, and climate change impacts.^6^^,^^7^

Cyanobacteria, as prominent photoautotrophs, are widely distributed in glacial habitats,^8^ particularly on glacier surfaces.^9^^,^^10^^,^^11^ They often serve as primary colonizers and play critical roles in biogeochemical cycling through oxygenic photosynthesis,^2^ carbon and nitrogen fixation,^12^ and chemical weathering of minerals.^13^ Additionally, their dark pigments reduce surface albedo, accelerating melt processes.^14^ By producing extracellular polymeric substances (EPS), Cyanobacteria can stabilize and improve habitat conditions, thereby influencing the stability of microbial communities.^15^

Most existing research on glacial Cyanobacteria has focused on cryoconite holes that are small, water-filled depressions on the glacier surface containing a mixture of mineral particles, organic matter, and microorganisms.^16^^,^^17^^,^^18^ Cryoconite holes provide a relatively stable microhabitat with higher nutrient availability and more liquid water compared to the surrounding ice and snow.^19^^,^^20^ However, the microbial communities in surface snow and ice differ significantly from those in cryoconite holes due to the more extreme and variable environmental conditions, including lower temperatures, less liquid water, and higher UV exposure.^2^^,^^21^ These communities are the first to be exposed to atmospheric inputs and surface melt processes, playing a key role in nutrient cycling and primary production in glacier ecosystems.^22^^,^^23^^,^^24^ Therefore, research on microbial communities in surface snow and ice is crucial for understanding the broader ecological processes of glacial habitats.

While the taxonomy and ecological roles of Cyanobacteria in glaciers have been well documented,^8^^,^^25^^,^^26^ few studies have examined their role in the dynamics and assembly of microbial communities, especially in surface snow and ice habitats. We hypothesize that Cyanobacteria dominate microbial communities on glacier surfaces and play an irreplaceable role in microbial community assembly.

**Figure 1:**
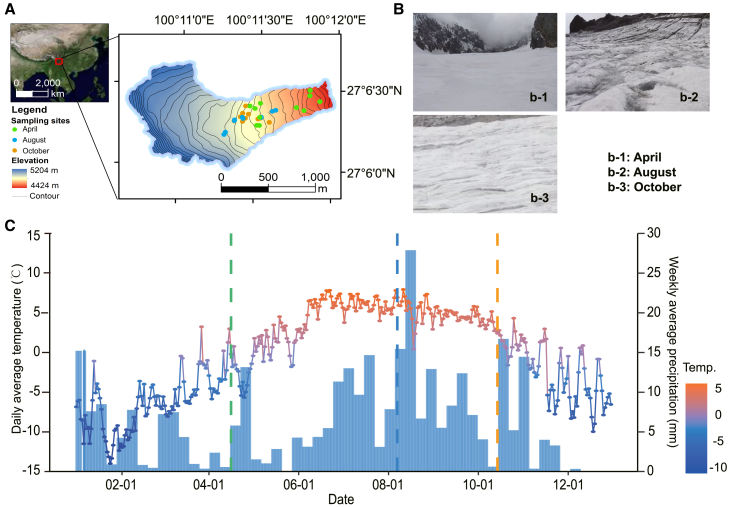
Overview of the research area (A) Geographic location and distribution of sampling points for Baishui Glacier No.1. (B) Photographs of glacier surfaces at different sampling times. (C) Annual temperature and precipitation changes in Baishui Glacier No.1.

To address this knowledge gap, we investigated microbial communities on the surface of Baishui Glacier No. 1 during different periods (Figure 1). Using quantitative PCR and high-throughput sequencing of 16S rRNA and 18S rRNA genes, we analyzed microbial abundance and community structure. Our study aims to elucidate the dynamics of microbial communities on glacier surfaces and to explore the specific role of Cyanobacteria in these processes.

## Results

### Temporal variation in microbial communities on glacier surfaces

Based on the combined abundance information from the three periods, the overall abundance of the microbial communities showed a continuous increase during the melt period. The average abundance increased from 1.8×10^4^ copies/ml in the accumulation period to 2×10^8^ copies/ml in the melt period and then reached an average of 3.8×10^8^ copies/ml in the late melt period (Figure S1). Cyanobacteria, Proteobacteria, Actinobacteriota, Deinococcus-Thermus, Bacteroidota, and SAR phylum (mainly composed of eukaryotic algae) were the six most abundant groups in the microbial community on the glacier surface, accounting for >95% of the microbial community abundance. In the accumulation period, members of Phragmoplastophyta and Ascomycota constituted over 80% of the glacier surface microbial community abundance. After the transition from the accumulation period to the melt period, members of six dominant phyla dominated the microbial community. Their relative abundances were as follows: Proteobacteria (23.91%), Bacteroidota (18.60%), SAR phylum (16.14%), Actinobacteriota (13.98%), Deinococcus-Thermus (11.97%), and Cyanobacteria (10.48%). In the late melt period, Cyanobacteria became the most abundant group in the community, accounting for 28.42% of the abundance. The abundances of the remaining five dominant phyla were as follows: Proteobacteria (23.98%), Deinococcus-Thermus (18.02%), Actinobacteriota (12.36%), SAR phylum (6.77%), and Bacteroidota (6.62%). At the genus level, the microbial community in the accumulation period was dominated by unclassified genera belonging to the class Embryophyta (Figure S2). By the melt period, several bacterial genera had become more prominent, including *Deinococcus*, *Polaromonas*, *Pseudonocardia*, *Hymenobacter*, and unclassified genera belonging to family Leptolyngbyaceae and class Chrysophyceae. In the late melt period, genera belonging to family Leptolyngbyaceae became abundant, while other genera, such as *Polaromonas* and *Deinococcus*, also maintained a significant presence.

**Figure 2:**
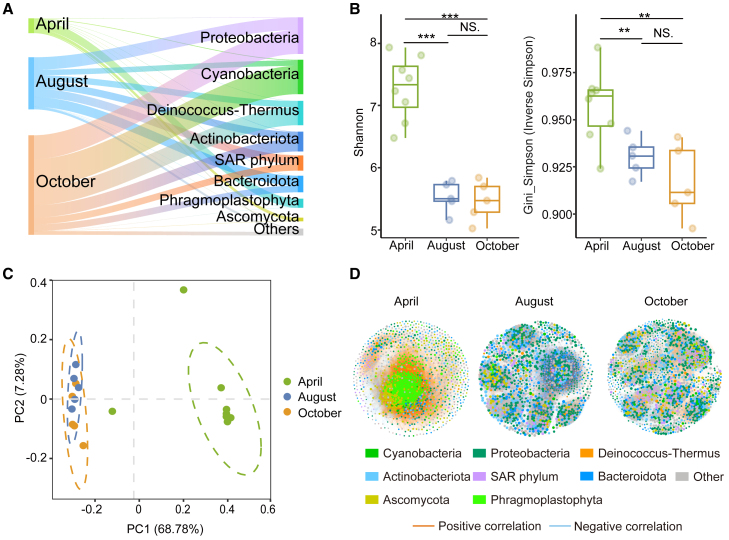
Temporal variation in microbial communities on glacier surfaces (A) Composition changes of microbial communities in glacier surface habitats during three periods. Flow indicates the absolute abundance. For April, abundance values were scaled by multiplying by 500 to enhance readability. (B) Differences in alpha diversity indices of microbial communities during different periods. Data are represented as boxplots overlaid with jittered points. The box displays the first quartile (Q1), median, and third quartile (Q3), while the whiskers extend to the minimum and maximum values within 1.5 times the interquartile range (IQR). Individual points represent data distribution, with jittering applied to prevent overlap. The significance of the *t* test is marked; “∗” indicates *p* < 0.05, “∗∗” indicates *p* < 0.01, “∗∗∗” indicates *p* < 0.001, “NS” indicates *p* > 0.05. (C) Principal component analysis (PCA) of microbial communities. Different colors indicate samples from different periods. (D) Microbial networks constructed using SparCC for different periods. Only correlations with r > 0.6 and *p* < 0.05 and non-isolated vertices were retained. OTUs are colored according to the phylum.

The diversity of the microbial communities also showed significant variation with the change in periods. The alpha diversity indices significantly decreased from the accumulation period to the melt period and remained stable from the melt period to the late melt period without significant changes (Figure 2B). The principal components analysis (PCA) was conducted to analyze beta diversity, and the principal coordinates obtained explained 76.06% of the variation in all samples (Figure 2C). PCA presented a similar pattern to the α-diversity indices, with significant differences in community composition between the accumulation period and the melt period, whereas the community composition between the melt period and the late melt period was similar. Overall, Adonis analyses demonstrated significant differences in community structure among the three periods (R_adonis_ = 0.4115^∗∗∗^). The Venn diagram shows that the microbial community had the most specific OTUs in the accumulation period, which was significantly higher than those in the melt period (Figure S3). A total of 1031 OTUs (15.9%) were shared across all three periods, indicating a core microbial community present throughout the year. In the accumulation period, 2194 unique OTUs (56.4%) were identified. In the melt period, 75 unique OTUs (1.9%) were detected, while in the late melt period, 160 unique OTUs (4.1%) were found. Additionally, 271 OTUs (7.0%) were shared exclusively between April and August, 177 OTUs (4.5%) were common to August and October, and 391 OTUs (10.1%) were shared between April and October.

### Role of cyanobacteria in microbial communities of glacier surface habitats

**Figure 3:**
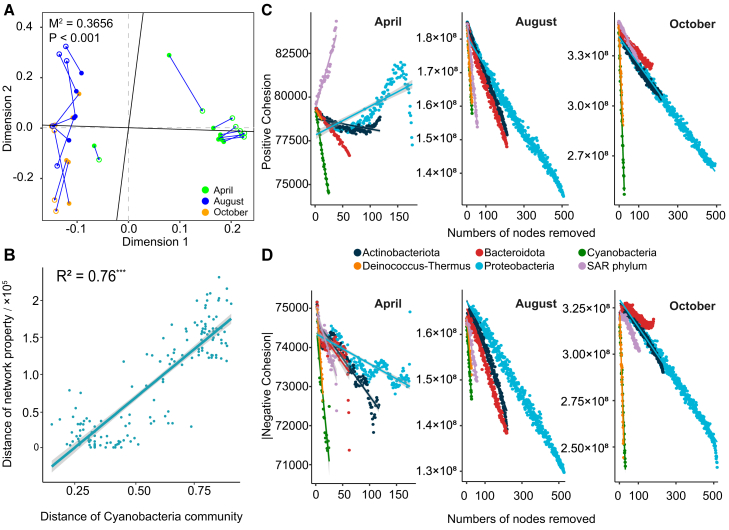
Role of Cyanobacteria in the microbial communities of glacier surface habitats (A) Procrustes analysis of the Cyanobacterial community and other microbial communities without Cyanobacteria. A smaller M2 value indicates a stronger correlation. (B) Linear regression of differences in Cyanobacteria composition and local microbial network properties (number of nodes, number of edges, degree, average path length, diameter, density, clustering coefficient, and modularity) (R2 = 0.76∗∗∗). (C) Changes in community stability (positive cohesiveness) caused by the removal of OTUs from each of the six dominant bacterial phyla. A smaller slope indicates that the phylum was more important for maintaining the stability of the community. (D) Changes in community stability (negative cohesiveness) caused by the removal of OTUs from each of the six dominant bacterial phyla.

The ecological role of Cyanobacteria in communities has been validated using various methods. Procrustes analysis revealed correlations between the community composition of the dominant phyla and the other phyla (Figures 3A and S4). The correlation between the Cyanobacteria and other microbial groups was highly significant (M^2^ = 0.3656^∗∗∗^), and the vector residuals did not show significant differences among the different periods (Figure S5).

The coherence index was used to analyze the stability of the microbial communities across dominant phyla. Compared with other phyla, the removal of Cyanobacteria OTUs resulted in the largest decrease in the positive coherence index and the networks in different periods exhibited a similar pattern (Figure 3C). In the negative coherence analysis, the removal of Cyanobacterial OTUs resulted in a decreasing trend similar to that of Deinococcus-Thermus, which was significantly lower than that of the other groups (Figure 3D). In the keystone species analysis, most of the keystone species in the accumulation period belong to Phragmoplastopphyta while Cyanobacteria and SAR phylum in the melting period. In the late melt period, Cyanobacteria accounted for 70% abundance of the keystone species (Figure S6).

Turnover rate analysis revealed that Cyanobacteria exhibited the highest turnover rates during the transition from the accumulation period to the melt period (72.97%) and from the accumulation period to the late melt period (73.26%), compared to other phyla. Conversely, the turnover rate from the melt period to the late melt period was the lowest (20.69%) (Table S1). In the accumulation period, the most abundant family in the Cyanobacteria was Chroococcidiopsaceae, which accounted for 87.85% of the Cyanobacteria abundance (Figure S7). From the accumulation period to the late melt period, the abundance of Leptolyngbyaceae became the most abundant family in the Cyanobacteria and the most abundant family in glacier surface microbial community, accounting for 24.85% of the total microbial community abundance.

Differences in the Cyanobacteria community were positively correlated with network topological properties (number of nodes, number of edges, degree, average path length, diameter, density, clustering coefficient, and modularity) (R^2^ = 0.76^∗∗∗^) (Figure 3B). This positive correlation indicates that greater differences in the Cyanobacteria community are associated with greater differences in the local microbial network. This finding underscored the strong association between Cyanobacteria and the structure of the local microbial community. Cyanobacterial abundance was also significantly correlated with various network topological properties, with the exception of density, the *p*-values of the correlations reached a significant level. Communities with higher Cyanobacteria abundance exhibited networks with a smaller number of nodes, fewer edges, lower degrees, shorter average path lengths, smaller diameters, higher densities, higher clustering coefficients, and higher modularity (Figure S8).

### Relationship between Cyanobacteria and other microorganisms

**Figure 4:**
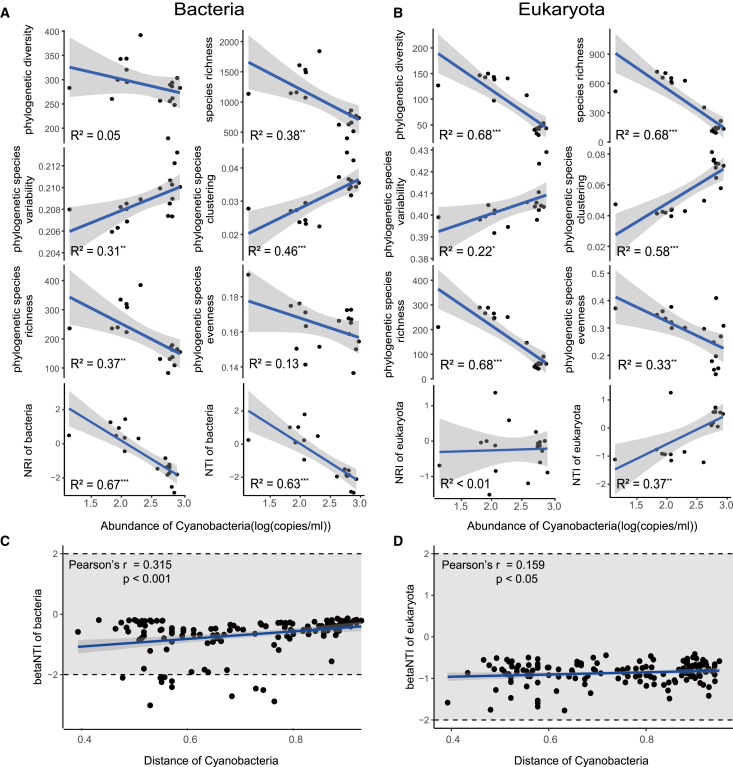
Correlation between Cyanobacteria and local microbial community phylogenetic structure and community assembly (A and B) Linear regression of Cyanobacteria abundance and PD(Phylogenetic diversity), SR(Species richness), PSV(Phylogenetic species variability), PSC(Phylogenetic species clustering), PSR(Phylogenetic species richness), PSE(Phylogenetic species evenness), NTI(Nearest taxon index) and NRI(Nearest relative index) of local Bacteria and Eukaryotes, “∗” indicates *p* < 0.05, “∗∗” indicates *p* < 0.01, “∗∗∗” indicates *p* < 0.001. (C and D) Linear regression and Pearson correlation between distance of Cyanobacteria and other microbial betaNTI(beta nearest taxon index).

Linear regression analysis revealed significant correlations between Cyanobacteria abundance and various microbial community metrics. As Cyanobacteria abundance increased, bacterial communities exhibited decreased phylogenetic diversity (PD) and species richness (SR), alongside increased phylogenetic species variability (PSV) and phylogenetic species clustering (PSC) (Figure 4A). Similarly, phylogenetic species richness (PSR) and phylogenetic species evenness (PSE) decreased (Figure 4A). Both the net relatedness index (NRI) and nearest taxon index (NTI) also decreased (Figure 4A). In eukaryotic microbial communities, increased Cyanobacteria abundance was associated with decreased phylogenetic diversity (PD) and species richness (SR), increased phylogenetic species variability (PSV) and phylogenetic species clustering (PSC), decreased phylogenetic species richness (PSR) and phylogenetic species evenness (PSE), but showed non-significant changes in NRI and increased NTI (Figure 4B). The distance of Cyanobacteria calculated by Sørensen distance was also significantly correlated with the betaNTI of other microbial communities (Figures 4C and 4D). This result suggests that Cyanobacteria play a significant role in the assembly of local microbial communities. The correlations between betaNTI of bacteria and eukaryotes and distance of Cyanobacteria were 0.315(*p* < 0.001), 0.159(*p* < 0.05).

In the prediction results from PICRUSt2, we identified 39 enzymes involved in nitrogen metabolism within the KEGG PATHWAY map00910. Subsequently, to explore the relationship between Cyanobacteria and the nitrogen metabolism of local other microorganism, we calculated the nitrogen metabolism multifunctionality of each sample using the averaging method, which did not include information of Cyanobacteria to avoid overfitting the abundance of Cyanobacteria with the nitrogen metabolic multifunctionality of other microorganisms. A significant correlation was found between the abundance of Cyanobacteria and nitrogen metabolism multifunctionality (R^2^ = 0.5, *p* < 0.001) of other local microorganisms (Figure S9).

**Figure 5:**
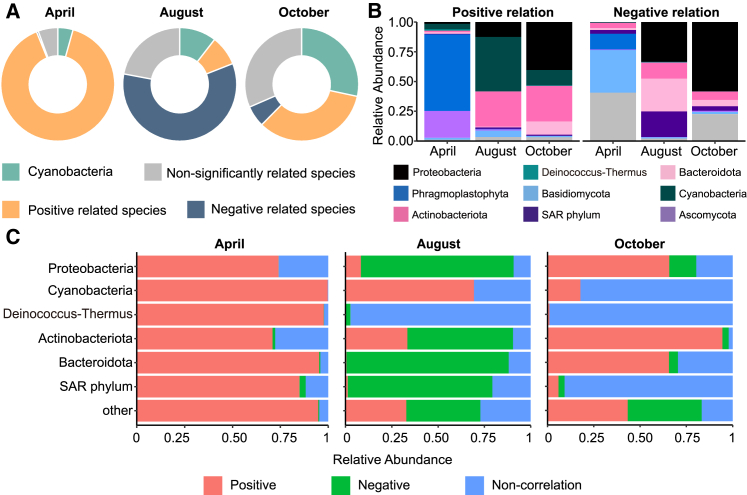
Relationship between Cyanobacteria and other microorganisms The species abundance for each period is calculated as the average across multiple sampling sites. (A) Abundance of Cyanobacteria and ZOTUs in which Cyanobacteria have established relationships in microbial communities. (B) Composition of microorganisms connected to Cyanobacteria at different periods. Relationships are determined by the positive or negative correlations of the edges extending from Cyanobacteria ZOTUs to connected nodes, classified by the phyla of the connected ZOTUs, highlighting associations established by Cyanobacteria within the network. (C) Relationship between other phyla and Cyanobacteria during different periods, indicating whether ZOTUs from each phylum are positively or negatively correlated with Cyanobacteria, emphasizing the impact of Cyanobacteria on different phyla.

The SparCC network analysis revealed significant correlations between ZOTUs of other phyla in the microbial community and ZOTUs of Cyanobacteria. In the accumulation period, most ZOTUs on the glacier surface (89.56%) exhibited positive correlations with Cyanobacteria, with only a small fraction showing negative correlations (0.005%) and a few ZOTUs having no significant correlation with Cyanobacteria (0.06%) (Figure 5A). In the melt period, negative correlations dominated the relationship between Cyanobacteria and other ZOTUs (58.90%), and a considerable number of ZOTUs were not significantly correlated with Cyanobacteria (22.05%). ZOTUs exhibiting positive correlations with Cyanobacteria accounted for only 0.09% of the community. In the late melt period, 31.56% of ZOTUs in the community were no longer significantly correlated with Cyanobacteria, 33.98% showed significant positive correlations, and 0.06% exhibited significant negative correlations with Cyanobacteria (Figure 5A).

In the accumulation period, the ZOTUs positively correlated with Cyanobacteria primarily belonged to Phragmoplastophyta and Ascomycota, whereas the few ZOTUs negatively correlated with Cyanobacteria were mostly from Basidiomycota and Phragmoplastophyta. In the melt period, Cyanobacteria exhibited positive correlations with Proteobacteria, Actinobacteriota, and ZOTUs of Cyanobacteria, whereas negative correlations were observed with Proteobacteria, Actinobacteriota, Bacteroidota, and SAR phylum. In the late melt period, ZOTUs positively correlated with Cyanobacteria belonged to Proteobacteria, Actinobacteriota, Bacteroidota, and Cyanobacteria, whereas ZOTUs negatively correlated with Cyanobacteria were predominantly Proteobacteria (Figure 5B).

## Discussion

### Differences of microbial communities in snow and ice on glacier surface in different periods

This study examined the microbial communities on the surface of Baishui Glacier No. 1 during the accumulation, melt, and late melt periods,^27^ revealing significant seasonal differences in microbial communities.

Combining the stress gradient hypothesis with our research findings, we found that the microbial community in the melt period suffered from relatively minimal environmental stress, as evidenced by the higher prevalence of competitive relationships in networks.^28^ Compared with the other two periods, the melt period is characterized by above-freezing temperatures and greater availability of liquid water on the glacier surface. Therefore, subfreezing temperatures and limited liquid water may be major limiting factors for microbial colonization of glacier surfaces, as reported in previous studies.^29^^,^^30^

Furthermore, based on the microbial community characteristics in different period, the microbial communities exhibited different responses to long- and short-term environmental stress. In the accumulation period, the microbial community faced long-term subfreezing temperature and liquid water limitations (Figure 1C). During the accumulation period, significant differences in community structure and composition were observed compared to the melt period. Consequently, long-term sub-freezing temperatures and liquid water scarcity exert pressure on microbes that deposited to the glacier, leading to relatively lower microbial activity on the glacier surface. Microorganisms that are unable to adapt to prolonged liquid water limitation may enter a dormant state^31^ or be environmentally filtered.

In the late melt period, the glacier surface experiences fluctuating diurnal temperatures near the freezing point, leading to short-term low temperature and liquid water limitations. Although there were no significant differences in community structure and composition between the melt period and the late melt period (Figures 1B and 1C), significant differences were observed in the microbial network (Figure S10). We propose that short-term liquid water limitations may have a relatively minor impact on the taxonomic-level responses of microbial communities but significantly affect species interactions within the community. Therefore, we suggest that microbial cooperation within communities is a crucial mechanism for resisting short-term environmental stress on glacier surfaces, as evidenced by the significant impact of liquid water limitations on microbial interactions despite stable community structure and composition.

### Development and dominance of Cyanobacteria in glacier surface microbial community

During the melt period, Cyanobacteria became the most abundant phylum on the glacier surface, accounting for one-third of the microbial abundance in the late melt period (Figure 2A). This phenomenon indicates that the environmental conditions during the accumulation period are less conducive for Cyanobacteria growth and reproduction than those during the melt period.

Cyanobacteria are widely distributed in glacier habitats^8^ and have been reported to have a variety of adaptation and tolerance mechanisms. These mechanisms include a high rate of organic nitrogen uptake in oligotrophic environments,^32^ specialized photosynthetic mechanisms,^33^ tolerance to intense sunlight and UV radiation, and systems for the dissipation of excess energy.^34^^,^^35^^,^^36^ These characteristics may form the basis for Cyanobacteria survival on glacier surfaces during the melt period.

The significant abundance of Cyanobacteria also suggests the success in competing with other microorganisms, especially eukaryotic algae. Microbial networks indicated that microorganisms were the most active and exhibited significant interspecies competition in the melt period (Figure 2D), during which Cyanobacteria maintained significant internal mutualistic relationships. Cooperation among individuals within a population can promote competition between populations,^37^ which has been experimentally verified in Cyanobacteria^38^ and may be an important factor in the competitive advantage of Cyanobacteria in glacier surfaces.

Additionally, Cyanobacteria have been reported to use other mechanisms to compete with other species, including the production of antibiotics,^39^ bioactive natural products such as toxins, carbon-concentrating mechanisms (CCMs), and optimization of photon capture efficiency for photosynthesis.^40^ These strategies, particularly CCMs^41^ and toxins,^42^^,^^43^ may play significant roles in their competition with eukaryotic algae. The genome may also be a crucial factor in the competitive advantage of Cyanobacteria. Their smaller genomes compared to eukaryotic algae make them more adaptable to extreme environments.^44^ Cyanobacteria genomes contain many genes derived from horizontal gene transfer (HGT)^45^ and a rich array of metabolic genes that can adapt their genomic functional composition to changes in environmental conditions on the glacier surface, enhancing ecological adaptability.^46^

Turnover rate analysis revealed that Cyanobacteria exhibited the most significant response to environmental changes during the early melt period and maintained a stable composition in the later period (Table S1). Cyanobacteria on the glacier surface during the melt period were mainly classified as Leptolyngbyaceae (Figure S7). Previous research indicates that Leptolyngbyaceae has been identified in various environments, including hot springs,^47^ freshwater,^48^ tropical grasslands,^49^ oceans,^50^ caves,^51^ saline lands,^52^ as well as soils and glaciers in polar regions.^13^ Recent research in polar lakes based on metagenome-assembled genomes(MAGs) found that Leptolyngbyaceae possess several genetic adaptations that may facilitate their survival on glacier surfaces.^53^ Although their presence on glaciers globally is well documented, their survival mechanisms for persisting on glaciers remain unclear.

### Influence of Cyanobacteria on local microbial community on glacier surfaces

Procrustes analysis indicated a significant association between the composition of Cyanobacteria and other microorganisms, surpassing that of the other dominant phyla (Figures 3A and S4). In addition, Cyanobacteria were significantly related to the phylogenetic diversity (Figures 4A and 4B) and community assembly (Figures 4C and 4D) of local microorganisms. These observations suggest that as Cyanobacteria adapt to changes in glacier surface habitats, they may also simultaneously affect biodiversity changes in other microbial groups.

Laboratory studies have shown that Cyanobacteria can alter the composition of microbial communities through cellular components released during growth and decay under intense light conditions.^54^ Despite the absence of visible cryoconite in all of our samples, similar phenomena have been observed in cryoconite, which are usually formed and dominated by Cyanobacteria.^13^^,^^16^ Microbial network analysis further supports this finding. Cyanobacteria significantly contributed to community stability, as evidenced by the rapid collapse of network stability when their nodes were removed (Figures 3C and 3D). Their relative abundance as keystone species increased, highlighting their crucial role during the melt period(Figure S6).^55^^,^^56^

Linear regression analysis revealed significant correlations between Cyanobacteria abundance and various microbial community phylogenetic index. The decrease in phylogenetic diversity (PD) and species richness (SR) in both bacterial and eukaryotic communities suggests that higher Cyanobacteria abundance correlates with reduced overall community diversity. Decreased community diversity may result from allelopathic effect, where Cyanobacteria produce compounds that inhibit the growth of other microorganisms.^57^^,^^58^ Alternatively, Cyanobacteria may provide an organic substrate for glacier surface habitats,^59^ influencing microbial community diversity through a developed food web based on the availability and diversity of this energy source.^60^^,^^61^ For example, by the late melt period, ZOTUs significantly associated with Cyanobacteria consisted mainly of predatory protists (Figure S12). The increase in phylogenetic species variability (PSV) and phylogenetic species clustering (PSC) indicates that remaining species are more phylogenetically diverse and clustered, possibly because Cyanobacteria create niche conditions that favor certain phylogenetic groups over others. Microbial network analysis further supports these findings(Figure S8).^13^^,^^62^ Communities with higher Cyanobacteria abundance had smaller scale, higher density, and greater modularity in their microbial networks. This implies that Cyanobacteria contribute to a more interconnected and stable community structure, potentially by forming mutualistic relationships with other microorganisms.

However, the contrasting trends in NRI and NTI between bacterial and eukaryotic communities warrant further discussion. In bacterial communities, both NRI and NTI decreased with increasing Cyanobacteria abundance, indicating a more phylogenetically dispersed community structure. This may be due to the strong competitive abilities of Cyanobacteria, which can exclude closely related bacterial taxa, thus promoting greater phylogenetic dispersion. Toward the late melt period, bacteria significantly associated with Cyanobacteria mainly included Actinobacteriota, *Hymenobacter*, *Granulicella*, and *Brevibacterium*, likely connected through diverse metabolic pathways such as UV and radiation adaptation,^63^^,^^64^ secondary metabolite production,^65^^,^^66^ extracellular polysaccharide production,^67^ and amino acid metabolism^68^^,^^69^(Figure S11).

In contrast, the non-significant change in NRI and significant increase in NTI in eukaryotic communities suggest a different mechanism. The increase in NTI indicates that closely related eukaryotic taxa are more prevalent, potentially due to facilitative interactions or shared adaptive traits that allow them to coexist with Cyanobacteria. The composition of eukaryotes significantly associated with Cyanobacteria exhibited marked changes. By the late melt period, the Cyanobacteria-associated eukaryotes were predominantly composed of predatory protists, such as Thecofilosea, Vampyrellidae, and Phyllopharyngea, as well as other eukaryotes dependent on Cyanobacterial nutrition, such as Chytridiomycota^70^ (Figure S12). In addition to the ability to fix carbon through photosynthesis, nitrogen metabolism may be the link between Cyanobacteria and local microorganisms especially in glacial habitats where nitrogen is a limiting factor.^21^ Our results found that high abundance of Cyanobacteria was accompanied by higher nitrogen metabolism multifunctionality of other local microorganisms.

In addition, during the late melt period, Cyanobacteria selectively maintained significant positive correlations with certain species in the community. These species are known to participate in the nitrogen cycle, such as *Polaromonas*,^22^^,^^71^^,^^72^ *Pseudonocardia*,^73^ *Ferruginibacter*,^74^ *Marisediminicola*,^75^ and others that are involved in denitrification. Murakami et al. observed relatively stronger denitrification activity in cryoconite on Asian glaciers, suggesting that nitrogen metabolism is likely a key link between Cyanobacteria and other microorganisms.^25^ Therefore, nitrogen metabolism may explain the symbiotic relationship between Cyanobacteria and other microorganisms,^76^^,^^77^ facilitating their colonization and survival.^78^^,^^79^

In conclusion, our study on the microbial community of Baishui Glacier No. 1 provides critical insights into the ecological responses and adaptations of microorganisms in extreme environments. We have highlighted the central role of Cyanobacteria, both as a dominant taxon and an ecosystem engineer, in influencing community structure and stability. High abundance of Cyanobacteria was associated with decreased diversity and increased phylogenetic clustering in both bacterial and eukaryotic communities. Microbial network analysis further revealed that communities with higher Cyanobacteria abundance had smaller, denser, and more modular networks, likely due to competitive exclusion and the selective establishment of mutualistic relationships by Cyanobacteria. These findings underscore their role as keystone species in glacier environments. In summary, as Cyanobacteria adapt to glacier surface conditions, they significantly influence biodiversity and community structure in other microbial groups. Notably, nitrogen metabolism appears to be a key link between Cyanobacteria and other microorganisms, as evidenced by the correlation between high Cyanobacteria abundance and enhanced nitrogen metabolism multifunctionality in local microbial communities.

### Limitations of the study

This study may overestimate the absolute abundance of eukaryotes due to their larger genome sizes and higher 18S rRNA gene copies compared to bacteria. This discrepancy likely underestimates bacterial abundance, including the ecological significance of Cyanobacteria. Another limitation is the inability to distinguish viable cells from non-viable ones, such as dormant spores or dead cells, particularly during the accumulation period.

## Resource availability

### Lead contact

Further information and requests for resources should be directed to and will be fulfilled by the Lead Contact, Binglin Zhang (zhangbl@lzb.ac.cn).

### Materials availability

This study did not generate new unique reagents.

### Data and code availability

• The 16S rRNA amplicon sequencing data generated in this study have been deposited in the Genome Sequence Archive in National Genomics Data Center, China National Center for Bioinformation/Beijing Institute of Genomics, Chinese Academy of Sciences (GSA: CRA012028) that are publicly accessible at https://ngdc.cncb.ac.cn/gsa.^80^^,^^81^
• This paper does not report original code.
• All relevant data supporting the findings of this study are available from the lead contact upon request.

## Acknowledgments

This work was funded by the 10.13039/501100001809National Natural Science Foundation of China (42071099, 42371156), the Key Project of Science and Technology of Gansu (23ZDFA010, 25JRRA491), and the Program of the State Key Laboratory of Cryospheric Science and Frozen Soil Engineering, Chinese Academy of Sciences (No. CSFSE-ZQ-2401).

## Author contributions

Conceptualization, B.Z.; methodology, Y.X. and M.W.; investigation, Y.X., Y.L., T.C., S.W., and B.Z.; formal analysis, Y.X. and S.W.; writing – original draft, Y.X.; writing – review and editing, Y.X., T.C., and B.Z.; funding acquisition, B.Z.; resources, T.C., G.L., G.Z., W.Z., X.C., and B.Z.; supervision, B.Z.

## Declaration of interests

The authors declare no competing interests.

## STAR★Methods

### Key resources table

**Table:** 

REAGENT or RESOURCE	SOURCE	IDENTIFIER
**Critical commercial assays**		

DNeasy PowerMax Soil Kit	QIAGEN, Corp., Germany	Cat#12988-10
AxyPrep DNA Gel Extraction Kit	Axygen Biosciences, Union City, CA, USA	Cat#MAG-FRAG-I-250

**Deposited data**		

Raw sequence data	This paper, Genome Sequence Archive in National Genomics Data Center (NGDC, https://ngdc.cncb.ac.cn/gsa)	GSA: CRA012027, CRA012028

**Oligonucleotides**		

338F	Shanghai Majorbio Bio-pharm Technology Co.,Ltd	5′-ACTCCTACGGGAGGCAGCAG-3′
806R	Shanghai Majorbio Bio-pharm Technology Co.,Ltd	5′-GGACTACHVGGGTWTCTAAT-3′
1380F	Shanghai Majorbio Bio-pharm Technology Co.,Ltd	5′-CCCTGCCHTTTGTACACAC-3′
1510R	Shanghai Majorbio Bio-pharm Technology Co.,Ltd	5′-CCTTCYGCAGGTTCACCTAC-3′

**Software and algorithms**		

fastp (0.19.6)	Chen^82^	https://github.com/OpenGene/fastp
FLASH (1.2.7)	Magoč et al.^83^	https://ccb.jhu.edu/software/FLASH/
USEARCH 11	Edgar^84^	http://www.drive5.com/usearch
RDP Classifier (2.2)	Wang et al.^85^	https://github.com/rdpstaff/classifier
R (4.0.3)	R Core Team^86^	https://cran.r-project.org
FastSpar (1.0.0)	Friedman et al.^87^ and Watts et al.^88^	https://github.com/scwatts/fastspar
Gephi (0.10.1)	Bastian et al.^89^	https://gephi.org/
igraph (R package)	Csárdi and Nepusz^90^	https://github.com/igraph/rigraph
vegan (R package)	https://rdrr.io/cran/vegan/	https://github.com/vegandevs/vegan
picante (R package)	Kembel et al.^91^	https://github.com/skembel/picante
picrust2	Douglas et al.^92^	https://github.com/picrust/picrust2

### Experimental model and study participant details

There were no experimental models involved in the study. Only snow and ice samples were collected and immediately frozen for this study.

### Method details

#### Samples collection

Samples were collected from the surface of Baishui Glacier No.1 (27° 06′ 16′′ N, 100° 11′ 44′′ E), with a depth of up to 20 cm, using high density polyethylene spatulas during three periods in 2021. A total of 30 samples were collected including 10 samples collected on April 16, which corresponded to the end of the accumulation period; 10 samples collected on August 7 which corresponded to the middle of the ablation period; 10 samples collected on October 14, which corresponded to the beginning of the accumulation period. In April, the glacier surface was covered by snow, so snow and bare ice samples were collected; in August and October there was only bare ice on the glacier surface, so bare ice samples were collected. Other than small stones visible to the naked eye, there were no other debris, such as cryoconite, in the snow and ice samples. Each sample was collected in triplicate, with the triplicates spaced approximately 1 meter apart. The samples were then uniformly homogenized into composite samples for the subsequent experiments. The samples were then placed in sterile Nasco Whirl-Pak bags. The samples were kept at -20°C during transportation and preserved until biodiversity analysis was performed. Local precipitation and temperature data were provided by the Yulong Snow Mountain Glacier and the Environment Observation and Research Station.

#### DNA extraction, amplification and sequencing

The melted water samples were filtered through a 0.22 μm filter, discarding any large stones at the bottom. The fine sediment and filter samples were combined and DNA was extracted using a DNeasy PowerMax Soil Kit (QIAGEN, Corp., Germany), following the manufacturer’s instructions. The quality and concentration of the DNA were determined using 1.0% agarose gel electrophoresis and a NanoDrop® ND-2000 spectrophotometer (Thermo Scientific Inc., USA). The V9 region of the eukaryotic small-subunit 18S rRNA gene was amplified using the primer set 1380F (5′-CCCTGCCHTTTGTACACAC-3′) and 1510R (5′-CCTTCYGCAGGTTCACCTAC-3′).^93^ The V3–V4 region of the bacterial 16S rRNA gene was amplified using the primer set 338F (5′-ACTCCTACGGGAGGCAGCAG-3′) and 806R (5′-GGACTACHVGGGTWTCTAAT-3′).^94^ All samples were amplified in triplicate. The PCR products were extracted from a 2% agarose gel, purified using an AxyPrep DNA Gel Extraction Kit (Axygen Biosciences, Union City, CA, USA) according to the manufacturer’s instructions, and quantified using a Quantus™ Fluorometer (Promega, USA). Sequencing was performed using an Illumina HiSeq2500 PE250 (Illumina Corp., USA). Raw FASTQ files were de-multiplexed using an in-house Perl script, quality-filtered using fastp version 0.19.6, and merged using FLASH version 1.2.7. For the 16S rRNA gene sequencing, fragments had an average length of 416 bp, with an average of 46,827 reads per sample. For the 18S rRNA gene sequencing, fragments had an average length of 136 bp, with an average of 20,239 reads per sample. Then, the optimized sequences were clustered into “zero-radius OTUs” (ZOTUs) using “-unoise3” commands in USEARCH 11. ZOTUs, also known as Exact Sequence Variants (ESVs) or Amplicon Sequence Variants (ASVs), represent unique DNA sequences without clustering based on a similarity threshold. This approach provides higher resolution for distinguishing closely related organisms and avoids the arbitrary nature of traditional similarity-based clustering thresholds. The ZOTUs table was manually filtered and the mitochondrial and chloroplast sequences of all samples were removed. The taxonomy of each ZOTUs representative sequence was analyzed by RDP Classifier version 2.2 against the 16S rRNA gene database (Silva v.138) using confidence threshold of 0.7. The raw sequence data reported in this paper have been deposited in the Genome Sequence Archive (GSA: CRA012027, CRA012028) in National Genomics Data Center(NGDC)^80^^,^^81^ and will be publicly available upon the article’s publication.

#### Quantitative real-time PCR

To evaluate the composition of microbial communities in different periods, a quantitative real-time PCR (qPCR) approach was used, using 16S and 18S rRNA genes as biomarkers. The same primers used for amplicon sequencing, including 338F/806R for 16S rRNA genes and 1380F/1510R for 18S rRNA genes, were employed for qPCR. SYBR Green I dye was used as the fluorescent detection agent and absolute quantification was performed using the standard curve method. qPCR reactions were carried out using 7300 and 750.0 Real-Time PCR Instrument Systems. The resulting qPCR data were expressed as the number of 16S or 18S rRNA gene copies per milliliter of sample to accurately reflect the absolute abundance of distinct microbial groups. Data from 16s and 18s rRNA gene amplicons were integrated with data from quantitative PCR.

#### Statistical analyses

All statistical analyses and graphs were performed using R 4.0.3 unless otherwise noted. To explore community stability and interspecific relationships, all ZOTUs were used to construct a SparCC network using FastSpar version 1.0.0.^87^^,^^88^ First, we calculated the initial correlation matrix using FastSpar, followed by generating 999 bootstrap samples to create a robust dataset for estimating correlation stability. These samples were analyzed in parallel to produce correlation and covariance matrices. Then, we calculated P-values for each correlation with 999 permutations, applying a final filter to retain only correlations with r > 0.6 and *P* value ≤ 0.05 before constructing the networks. Network graphs were generated using Gephi version 0.10.1.^89^ Network topological indices were calculated by the “igraph” R package, including numbers of nodes and edges, degree, average path lengths, diameter, density, clustering coefficient, and modularity. For each node, its within-module connectivity (Zi) and among-module connectivity (Pi) were calculated and used to classify keystone nodes in the network. Module hubs (Zi ≥ 2.5, Pi < 0.62), connectors (Zi < 2.5, Pi ≥ 0.62), and network hubs (Zi ≥ 2.5, Pi ≥ 0.62) were referred to as keystone nodes whereas other nodes were categorized as peripherals. Keystone nodes play a critical role in connecting different microbial groups or maintaining the stability of specific community modules. Cohesion was calculated to quantify the connectivity of microbial communities and evaluate the stability of microbial networks, including positive and negative cohesion values.^95^ The nodes of each of the six most abundant phyla were randomly removed to assess the impact of the six dominant taxa on community stability by comparing the decreasing cohesion trends. ZOTUs represented by nodes positively correlated with Cyanobacteria were identified as positively correlated species, whereas species represented by nodes negatively correlated with Cyanobacteria were identified as negatively correlated species. If a node was associated with more than one Cyanobacteria node, the group was divided according to the sum of the weights of all edges.

We chose the Shannon index and the Gini-Simpson index to represent alpha diversity, and were calculated using the “diversity” function (vegan package). The Shannon index accounts for both the richness and evenness of communities, providing insight into community complexity. The Gini-Simpson index (also known as the Inverse Simpson index) complements the Shannon index by focusing on community evenness and dominance.

To examine the impact of Cyanobacteria abundance on bacterial community structure, we calculated several diversity and phylogenetic metrics, including phylogenetic diversity (PD), species richness (SR), phylogenetic species variability (PSV), and phylogenetic species clustering (PSC). Additionally, we assessed phylogenetic species richness (PSR) and phylogenetic species evenness (PSE) to capture different dimensions of community diversity and structure. These phylogenetic metrics provide deeper insights by accounting for the evolutionary relationships within the community. All metrics were computed using the “picante” package in R. The betaNTI of bacterial and eukaryotic communities was calculated by “picante” package to characterize their community assembly mechanisms. The correlation between the six dominant phyla and their respective communities of other species was assessed by Procrustes analysis based on principal components analysis (PCA). Procrustes analysis is a statistical technique used to analyze the congruence between two configurations of data points by optimal superimposition, achieved through translation, rotation, and scaling operations, minimizing the sum of squared Euclidean distances between corresponding points. A smaller M^2^ value indicates a stronger correlation in the Procrustes analysis. Additionally, the residuals in Procrustes analysis represent the degree of dissimilarity between the two microbial communities. Lower residuals indicate stronger agreement between them, while higher residuals suggest greater differences.

Turnover rate refers to the rate at which microbial communities change over time. Turnover rates were calculated using pairwise comparisons between these periods, based on the presence or absence of OTUs within each phylum. Samples were grouped by period, and ZOTUs from the same period were combined by their presence or absence. We calculated and compared turnover rates for different phyla of microorganisms, with higher turnover rates indicating more drastic changes in the community. Cyanobacteria community similarities or dissimilarities (beta diversity) were calculated by the Sørensen distance; network topology index differences were calculated using Euclidean distance.

To explore the impact of Cyanobacteria on local community metabolism, nitrogen multifunctionality was calculated based on picrust2^92^ prediction and enzyme selection based on the KEGG(Kyoto Encyclopedia of Genes and Genomes) nitrogen metabolism map(map00910). The prediction results of 16S rRNA and 18S rRNA genes were merged with qPCR data for linear fitting analysis with the abundance of Cyanobacteria. Cyanobacteria were not included in the OTU table used for prediction to avoid overfitting caused by the nitrogen multifunctionality of Cyanobacteria themselves. We assessed nitrogen multifunctionality using averaging approaches. To obtain a quantitative multifunctionality index for each sample, we first normalized and standardized each of the evaluated nitrogen metabolism enzyme using the Z-score transformation. The Z-scores were then averaged to obtain a multifunctionality index for each sample.

### Quantification and statistical analysis

#### Alpha and beta diversity

The Shannon index and Gini-Simpson index were calculated for each composite sample using the diversity function in the vegan package. Differences of alpha diversity were assessed using two-sided t-tests by t.test function in stats package. Multiple comparisons were adjusted using the Benjamini-Hochberg false discovery rate (FDR) correction. Data are represented as boxplots overlaid with jittered points. The box displays the first quartile (Q1), median, and third quartile (Q3), while the whiskers extend to the minimum and maximum values within 1.5 times the interquartile range (IQR). Individual points represent data distribution, with jittering applied to prevent overlap. Beta diversity was assessed using Bray-Curtis distance and Sørensen distance, computed with the vegdist function in vegan. Adonis analyses demonstrated significant differences in community structure among the three periods by adonis function in vegan package.

#### Phylogenetic metrics and community assembly

In addition to alpha diversity, various phylogenetic metrics were computed, including Phylogenetic Diversity (PD), Species Richness (SR), Phylogenetic Species Variability (PSV), Phylogenetic Species Clustering (PSC), Phylogenetic Species Richness (PSR), and Phylogenetic Species Evenness (PSE). All calculations were performed with the picante package in R. Beta Nearest Taxon Index (betaNTI) was used to determine whether community assembly was deterministic or stochastic, with larger deviations from the null model suggesting a stronger deterministic process. The corresponding values and statistical significance are presented in the figures, figure legends, or results section.

#### Network construction and topological analysis

To investigate species relationships and overall community stability, SparCC correlation networks were constructed using FastSpar (version 1.0.0). A total of 999 bootstrap iterations were performed to assess the robustness of correlation coefficients. Only edges with correlation coefficients greater than 0.6 and p ≤ 0.05 were retained. The resulting networks were visualized in Gephi (version 0.10.1), and node degree, average path length, clustering coefficient, diameter, density, and modularity were calculated via the igraph package in R. Sørensen distance was used to measure community similarity, while Euclidean distance was employed to compare differences in network topology. Linear fitting is implemented using lm function in R.

Within each network, nodes were classified based on their within-module connectivity (Zi) and among-module connectivity (Pi): nodes with Zi ≥ 2.5 and Pi < 0.62 are defined as module hubs, those with Zi < 2.5 and Pi ≥ 0.62 are connectors, and those with Zi ≥ 2.5 and Pi ≥ 0.62 are network hubs. These three categories are collectively referred to as keystone nodes. Cohesion values based on positive and negative edges were computed to evaluate network stability. Random removals of nodes belonging to the six most abundant phyla were conducted to examine their influence on network stability.

#### Correlation analysis with cyanobacteria

In the co-occurrence network, nodes showing positive correlations with Cyanobacteria (correlation coefficient > 0.6 and p ≤ 0.05) were considered positively correlated species, while negatively correlated nodes were considered negatively correlated species (correlation coefficient < -0.6 and p ≤ 0.05). If a single node was correlated with multiple Cyanobacteria nodes, it was grouped according to the sum of all associated edge weights.

#### Turnover rate and community similarity

Turnover rate was calculated by comparing the presence or absence of ZOTUs at different sampling periods; higher turnover rates indicate more pronounced community changes.

#### Procrustes analysis

To evaluate the concordance between major dominant phyla and their associated communities, Procrustes analysis was conducted using Principal Components Analysis (PCA). The alignment between the two datasets was assessed using the procrustes function in vegan package, with 999 permutations applied to test statistical significance. The Procrustes M2 value and residuals were recorded, where smaller M2 values and residuals indicate a stronger similarity between the two community structures.

#### Nitrogen multifunctionality analysis

During functional prediction with PICRUSt2, Cyanobacteria sequences were excluded to avoid overfitting when examining the association between Cyanobacteria abundance and predicted nitrogen functions. KEGG orthologs (KOs) were selected based on the nitrogen metabolism pathway (map00910), and qPCR data were used to analyze the relationship between Cyanobacteria abundance and nitrogen functional potential. Each predicted enzyme activity was standardized by Z-score, and these scores were averaged to derive a nitrogen multifunctionality index. The correlation between the abundance of Cyanobacteria and nitrogen multifunctionality was assessed using the lm function in R.

#### Statistical reporting

All analyses adopted a significance threshold of p ≤ 0.05 adjusted using the Benjamini-Hochberg false discovery rate (FDR) correction. n = 18 represents the number of successfully sequenced samples. Specific statistical tests, p values, and effect sizes are documented in the figure legends or results text. Raw sequencing data have been deposited in the National Genomics Data Center (NGDC) (GSA: CRA012027, CRA012028). The statistical methods, software, and versions described above are consistent with the method details section, and further numeric details are available in the corresponding figures and results section.
