# Supplementary material for: Role of Cyanobacteria in the assembly and dynamics of microbial communities on glacier surfaces
Source: iScience. 2025 Feb 18;28(3):112061. doi: 10.1016/j.isci.2025.112061 (PMC11915163; doi:10.1016/j.isci.2025.112061)
Supplement: Document S1. Figures S1–S12 and Table S1 [file mmc1.pdf]

iScience, Volume *28*

Supplemental information

Role of Cyanobacteria in the assembly and dynamics of microbial communities on glacier surfaces

Yeteng Xu, Yang Liu, Tuo Chen, Shijin Wang, Guangxiu Liu, Gaosen Zhang, Wei Zhang, Minghui Wu, Ximing Chen, and Binglin Zhang

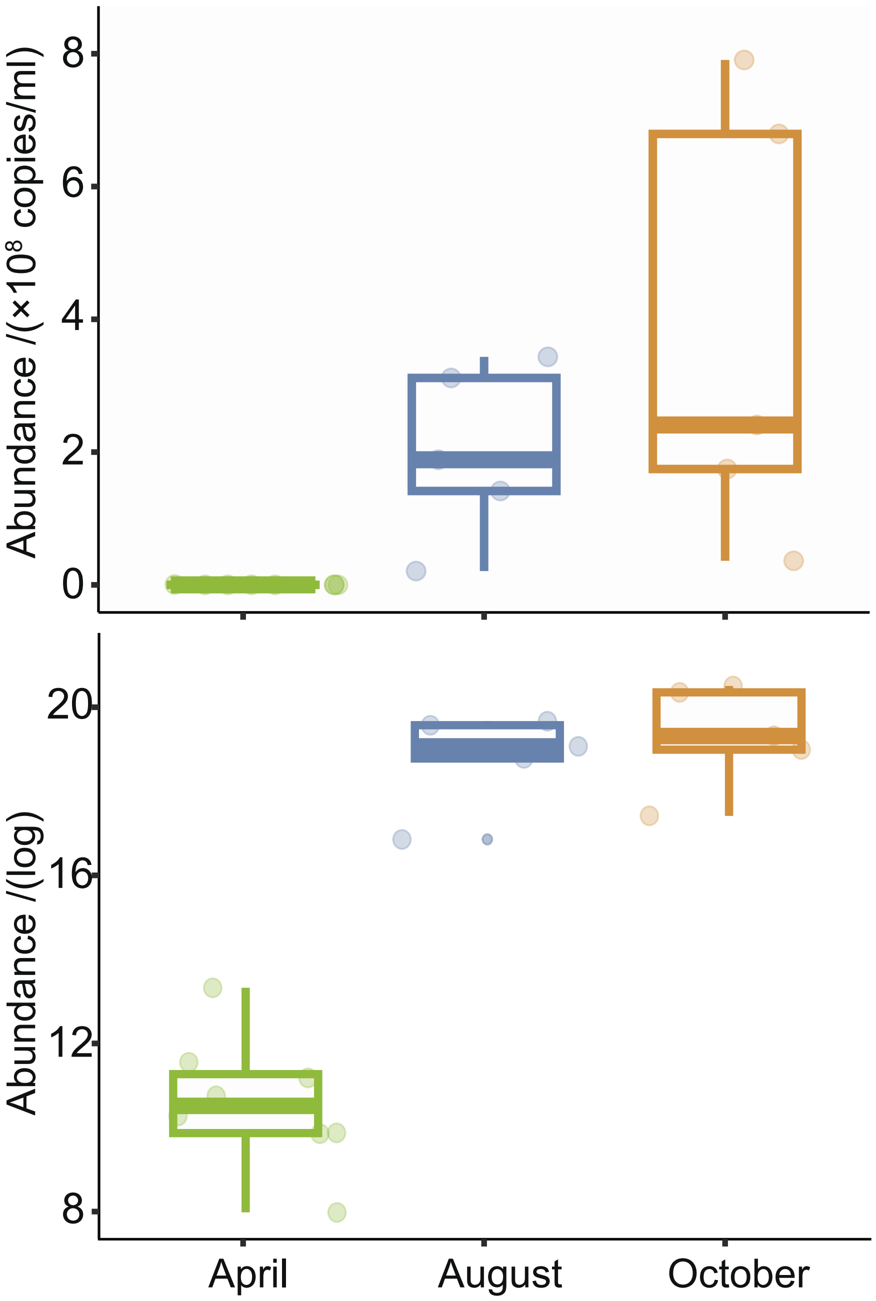

Figure S1. Absolute abundance of microbial communities on glacier surfaces in different periods, related to Figure 2.

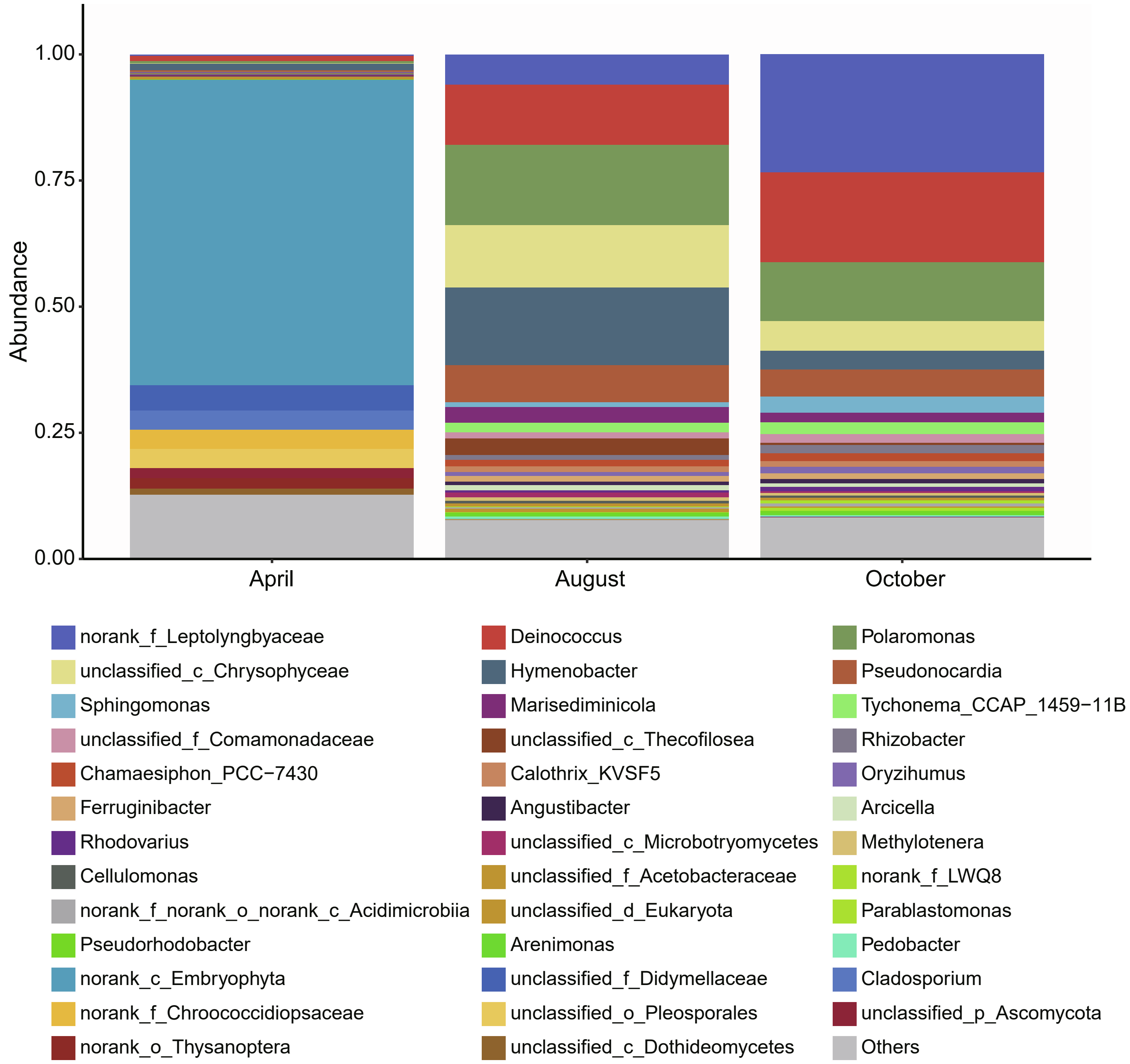

Figure S2. Genus composition of microbial communities at different periods, related to Figure 2.

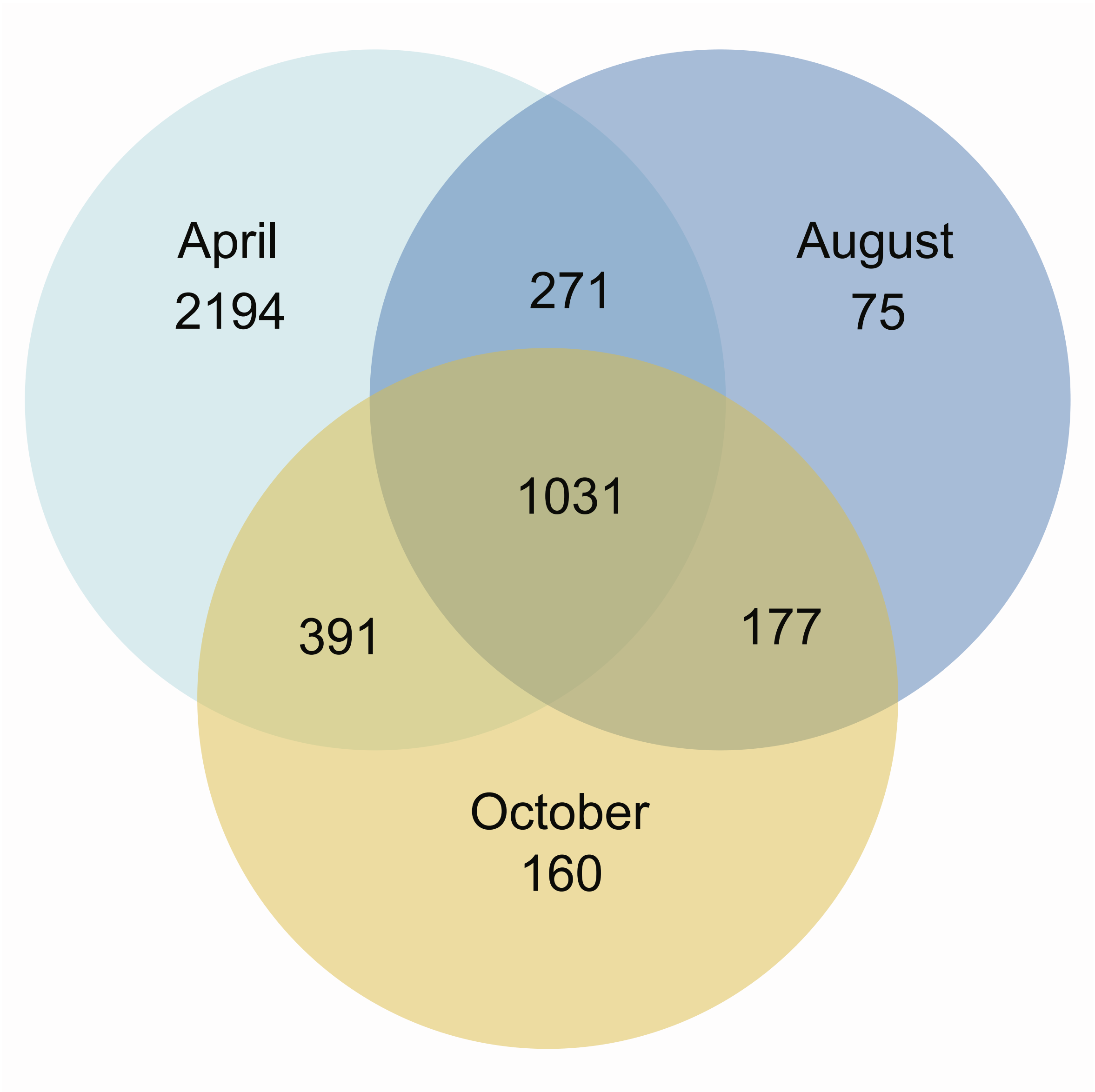

Figure S3. Venn diagram of shared and specific OTUs of microbial communities on glacier surfaces in different periods, related to Figure 2.

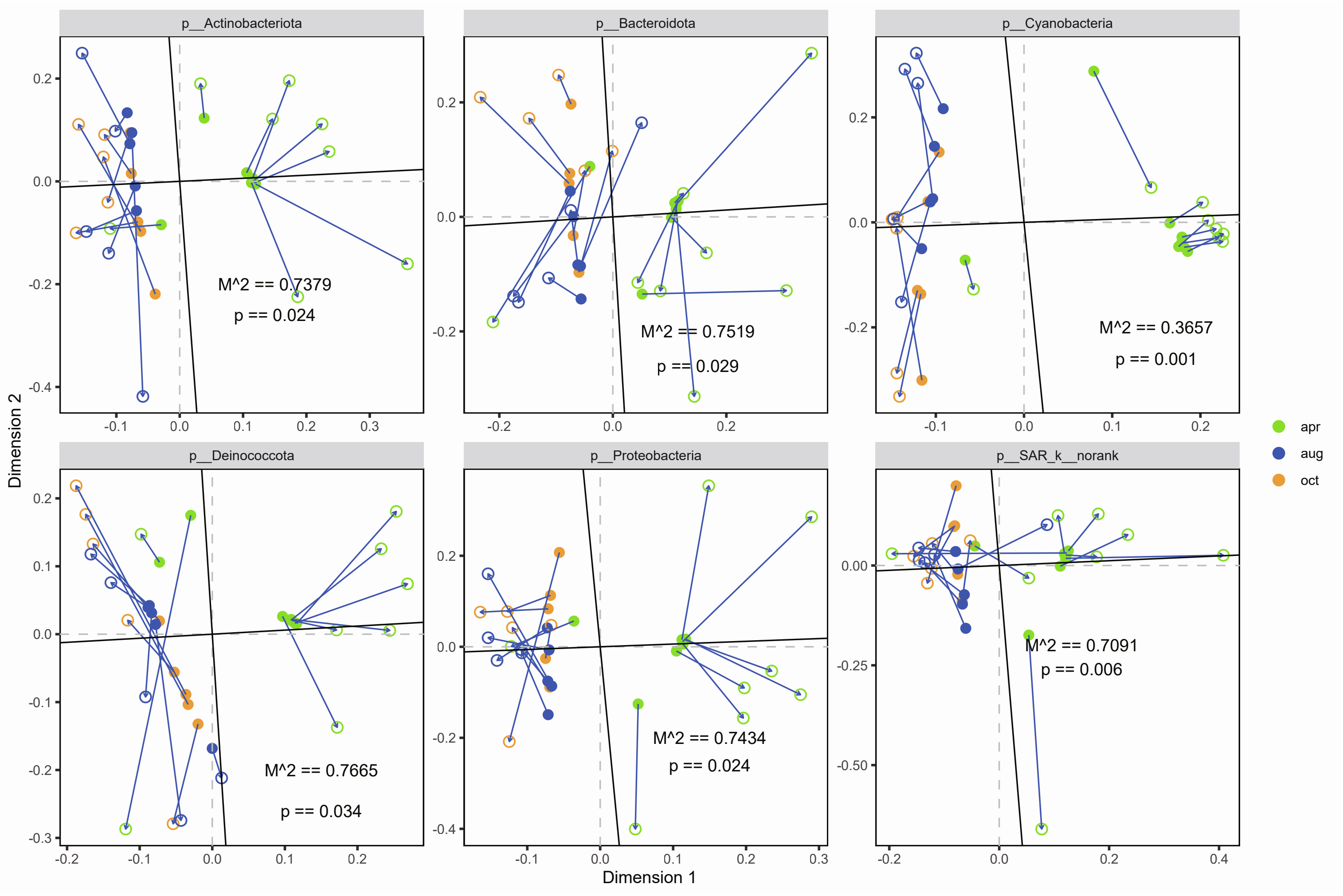

Figure S4. Procrustes analysis of each dominant phylum with corresponding other microbial communities, related to Figure 3.

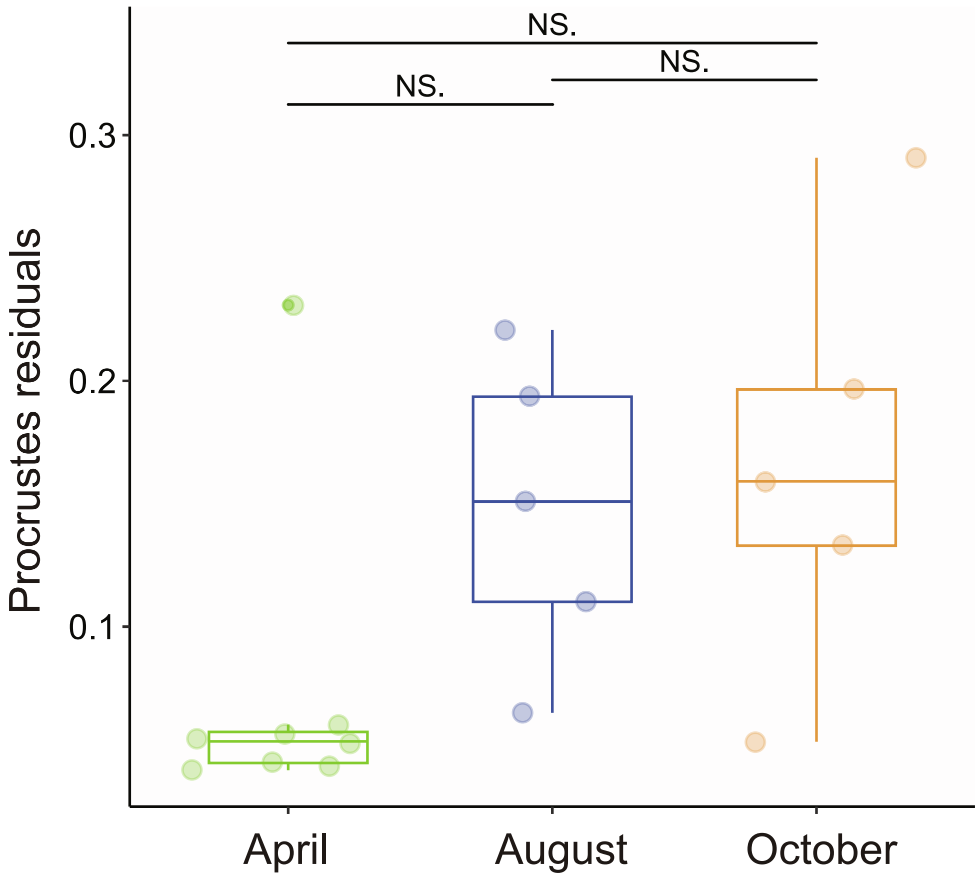

Figure S5. Residuals of Cyanobacteria paired with other microbial communities for each period in the Procrustes analysis, related to Figure 3.

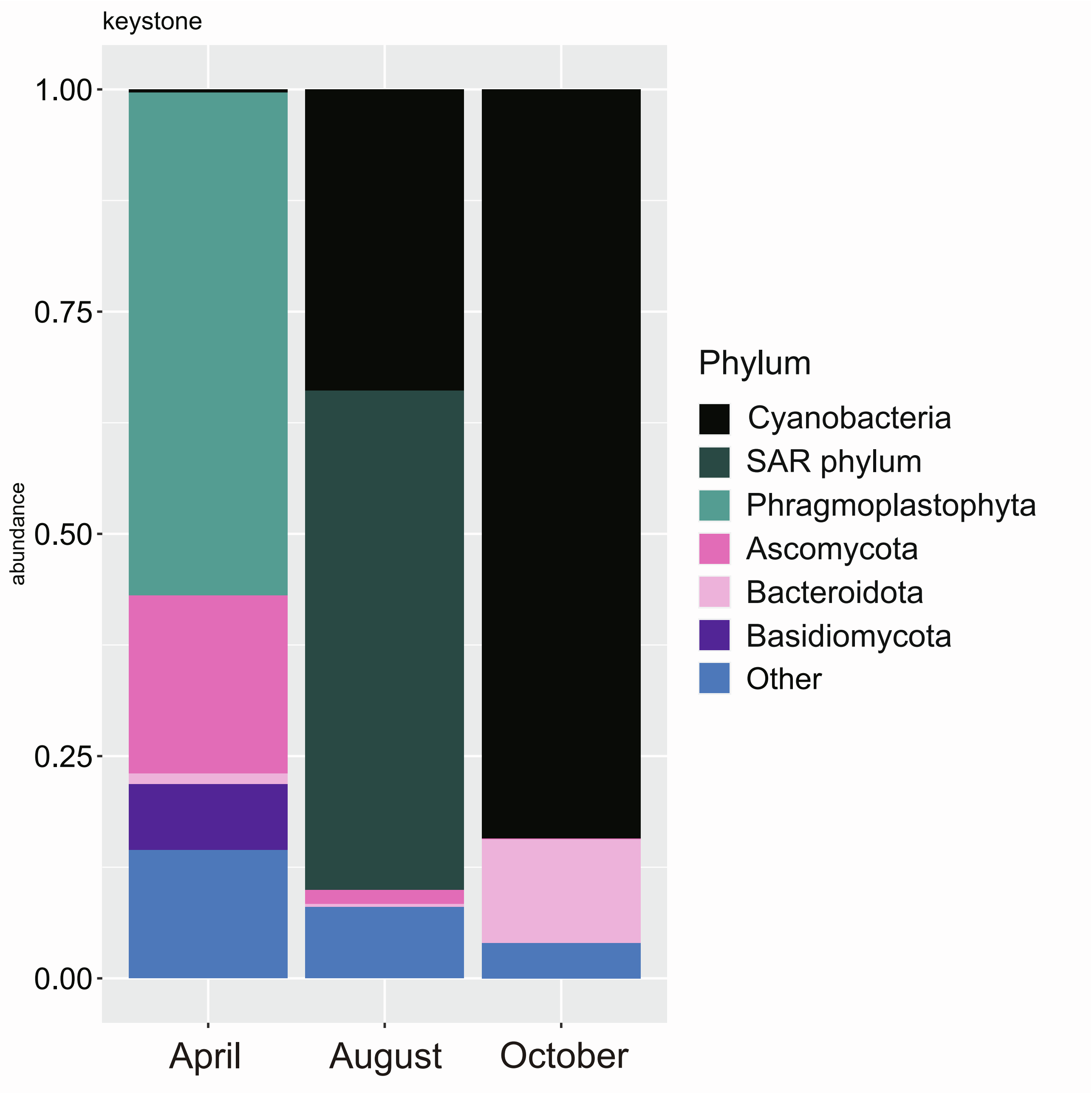

Figure S6. Species composition of the keystone species community on the surface of glaciers in different periods, related to Figure 3.

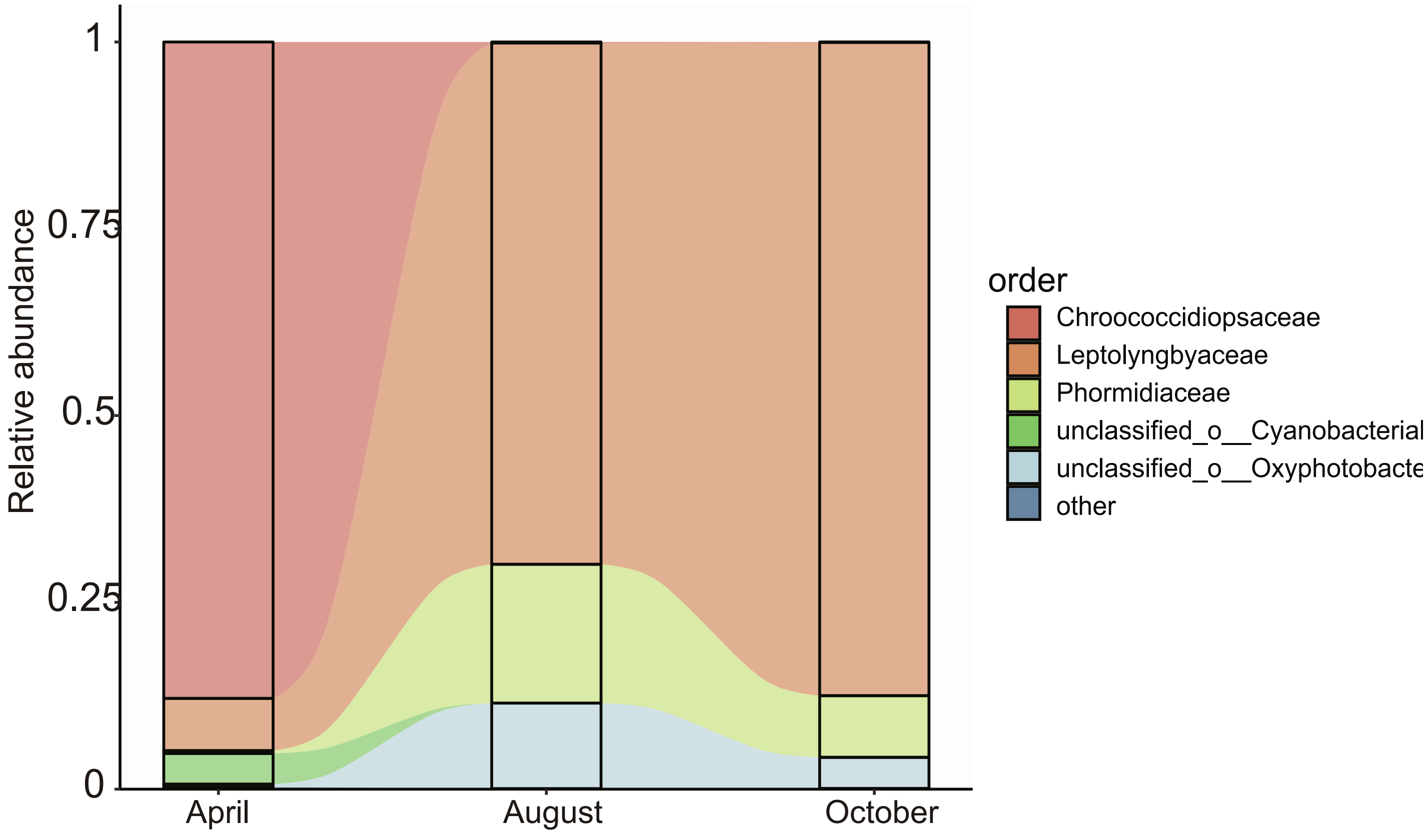

Figure S7. Changes in the order composition of Cyanobacterial communities in different periods, related to Figure 2.

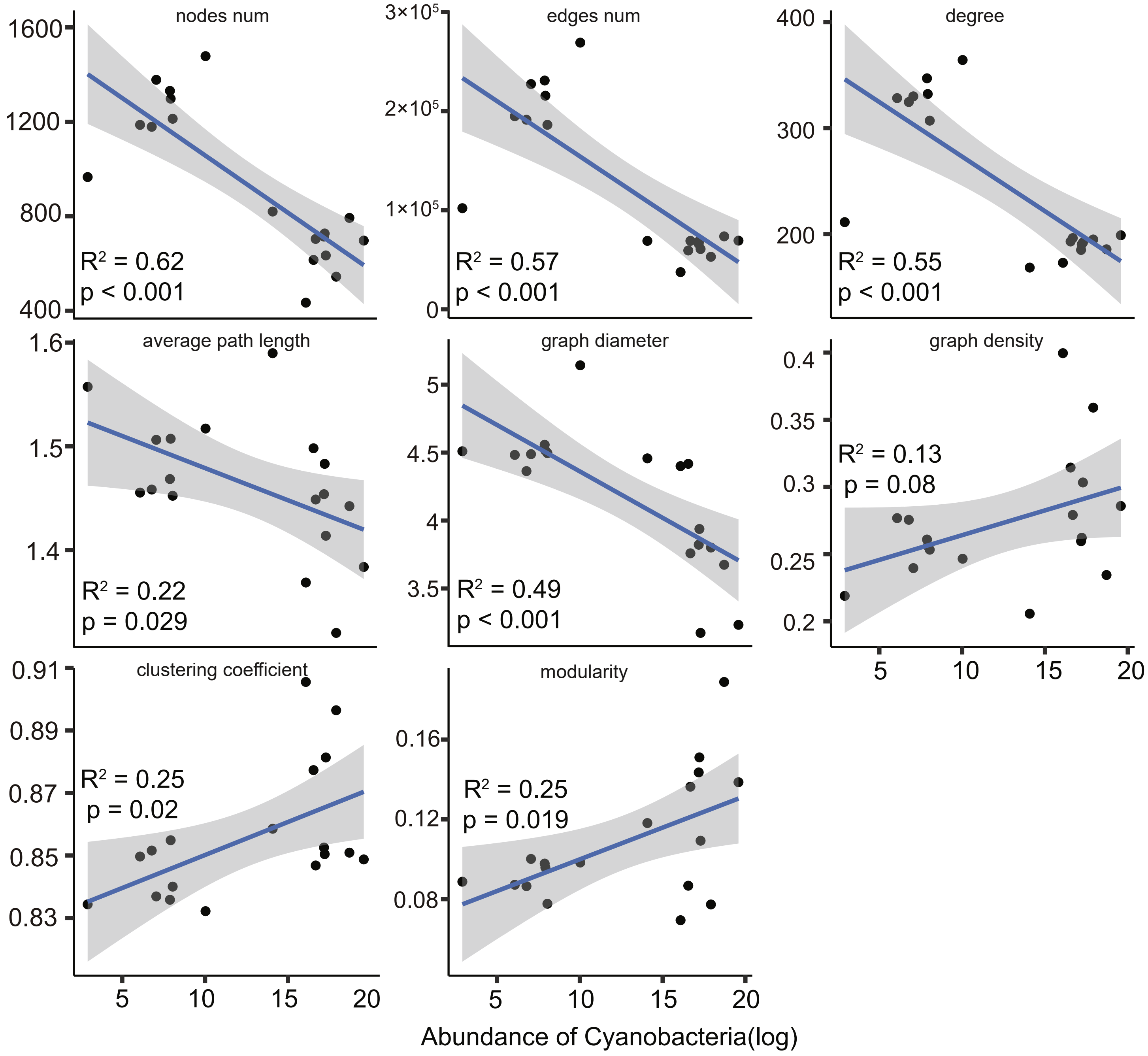

Figure S8. Linear regression of Cyanobacterial abundance(log) and local microbial network properties, related to Figure 3.

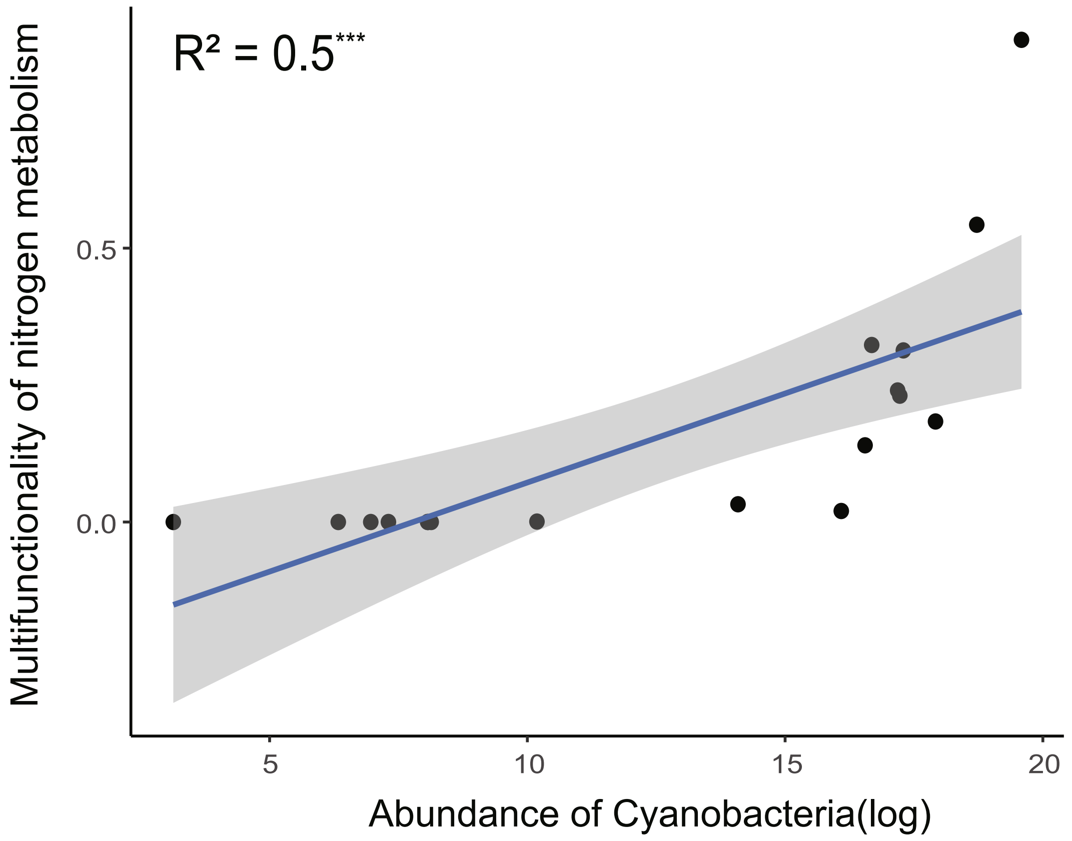

Figure S9. Linear regression of Cyanobacteria abundance and nitrogen metabolism multifunctionality of other local microorganisms, related to Figure 5.

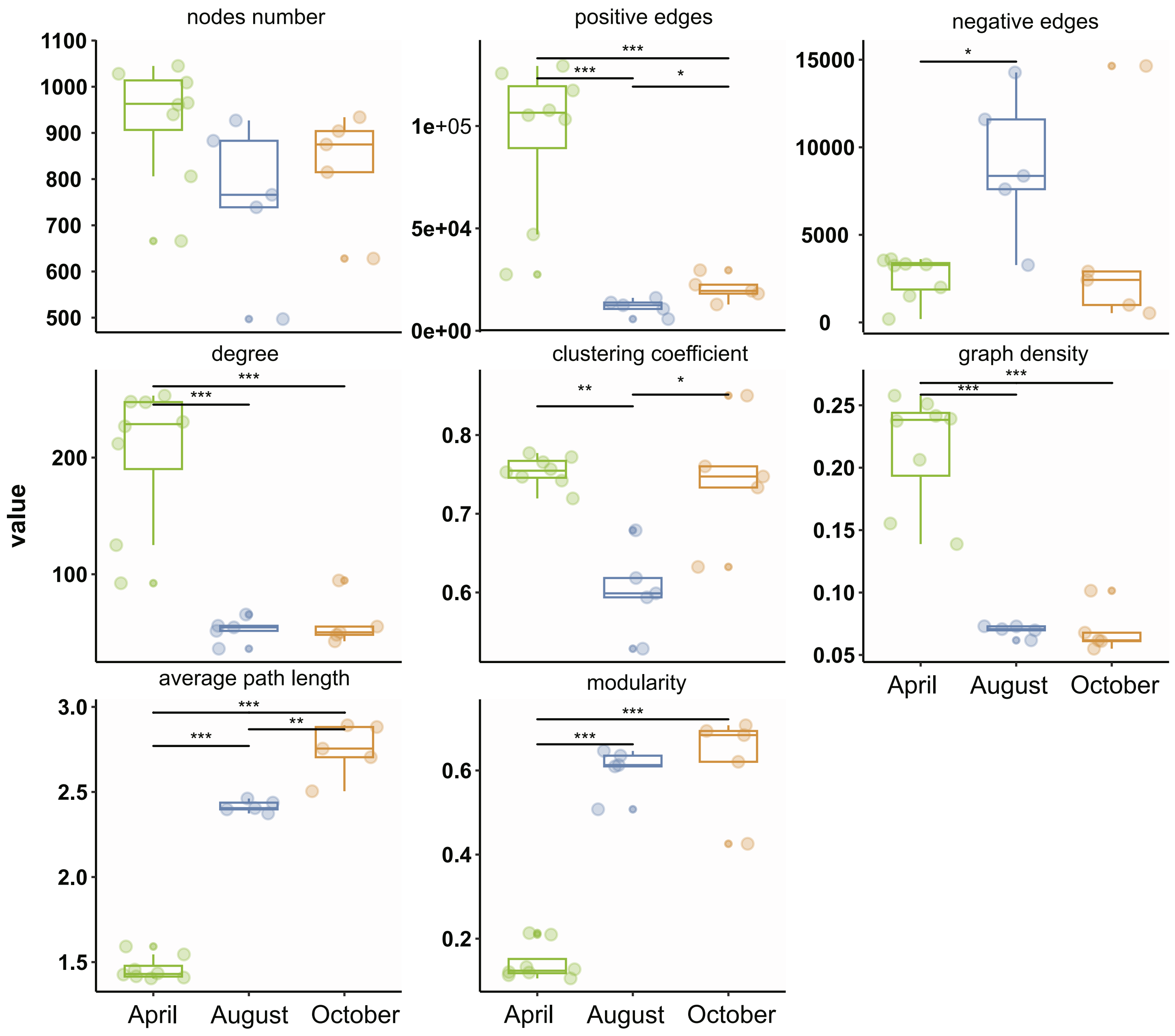

Figure S10. Differences in microbial network properties on glacier surfaces at different time periods, related to Figure 2.

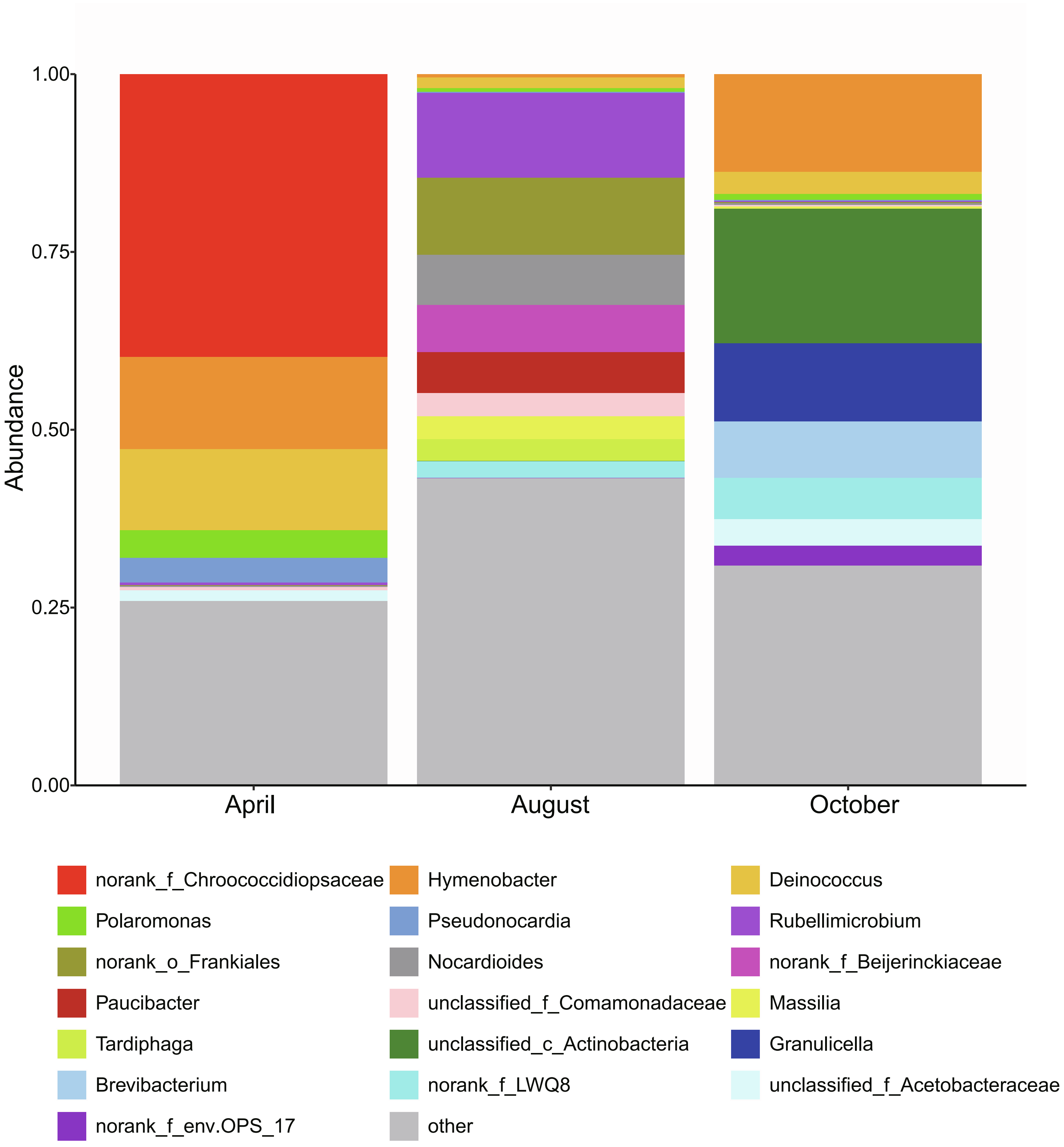

Figure S11. Composition of bacterial genera significantly related to Cyanobacteria, related to Figure 5.

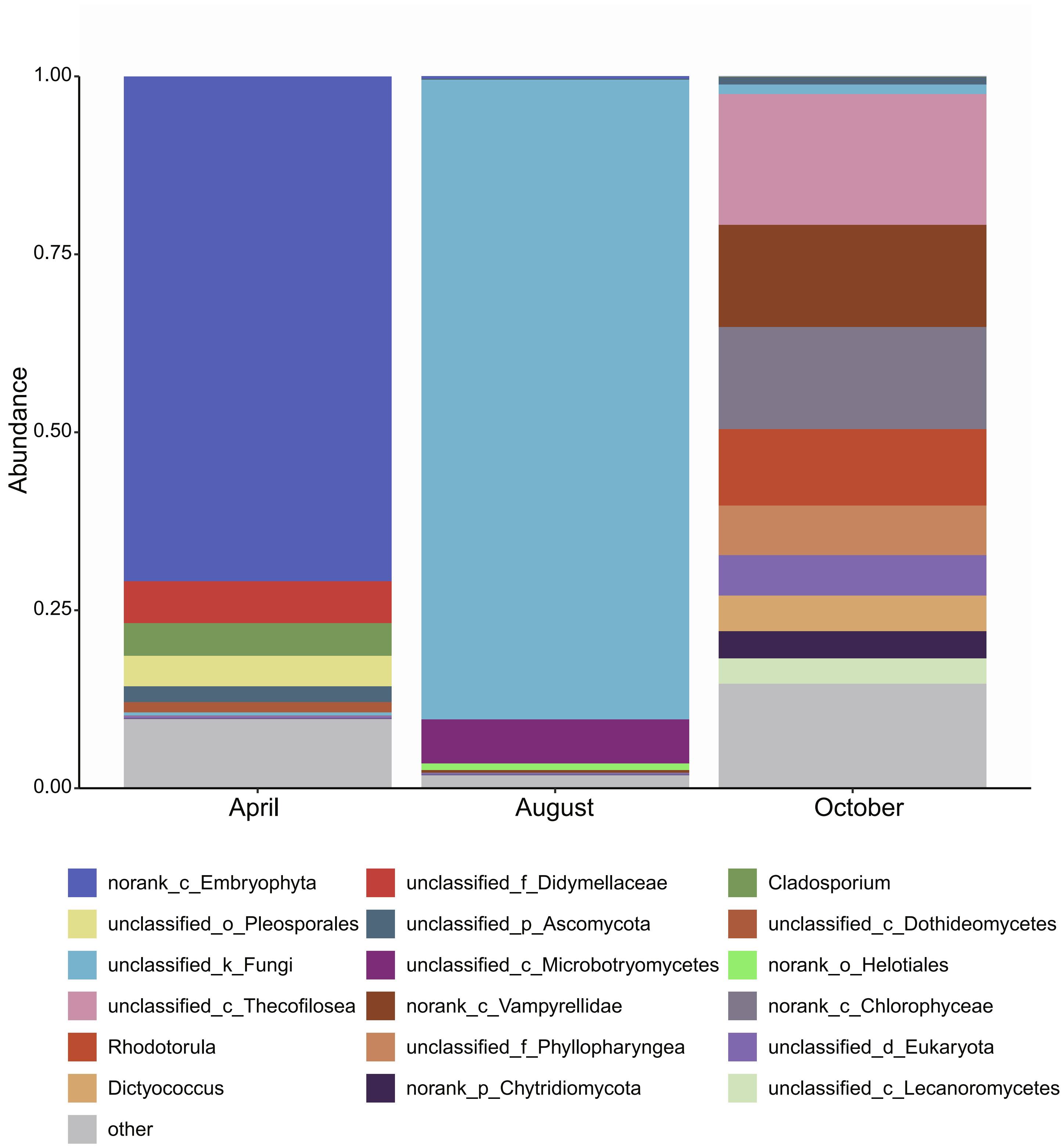

Figure S12. Composition of eukaryotic genera significantly related to Cyanobacteria, related to Figure 5.

Table S1. Turnover rates for dominant phyla and non-dominant phyla OTUs, related to Figure 2

	April to August	April to October	August to October
Proteobacteria	44.31%	45.12%	23.45%
Cyanobacteria	72.97%	73.26%	20.69%
Deinococcus-Thermus	54.35%	58.62%	31.91%
Actinobacteriota	51.29%	48.19%	28.16%
Bacteroidota	45.21%	50.35%	21.30%
SAR_k_norank	40.69%	35.42%	34.19%
other	59.37%	52.12%	31.22%
